# Formulæ Used in the Treatment of Tinea Capitis

**Published:** 1841-11

**Authors:** 


*Formulæ used in the Treatment of Tinea Capitis.*—The following are the formulæ commonly employed by M. Casenave in the treatment of this disease at the hospital of St. Louis:

*Ioduret of Sulphur Ointment*—Ioduret of sulphur, 1 scruple; lard, 30 scruples.

*Depilatory Ointment*—Subcarbonate of soda, 8 scruples; lime, 4 scruples; lard, 30 scruples.

*Pitch Ointment*—Citrine ointment, 15 scruples; pitch ointment, 30 scruples—Or, powdered pepper, 2 to 4 scruples; lard, 30 scruples.

The ointment is applied every evening; in the morning the head is washed with the following lotion:—Subcarbonate of potash, 8 scruples; distilled water, 500 scruples.—*Prov. Med. and Surg. Jour.*, August 14, 1841, from *Jour de Med. Pract.*, *Amer. Jour. Med. Sci.*

